# Advanced analytical methods to assess physical activity behavior using accelerometer time series: A scoping review

**DOI:** 10.1111/sms.14085

**Published:** 2021-11-01

**Authors:** Anne Backes, Tripti Gupta, Susanne Schmitz, Guy Fagherazzi, Vincent van Hees, Laurent Malisoux

**Affiliations:** ^1^ Physical Activity, Sport and Health Research Group Department of Population Health Luxembourg Institute of Health Strassen Luxembourg; ^2^ Competence Center for Methodology and Statistics Luxembourg Institute of Health Strassen Luxembourg; ^3^ Deep Digital Phenotyping Research Unit Department of Population Health Luxembourg Institute of Health Strassen Luxembourg; ^4^ Department of Public and Occupational Health Amsterdam Public Health Research Institute Amsterdam UMC Vrije Universiteit Amsterdam Amsterdam The Netherlands; ^5^ Accelting Almere The Netherlands

**Keywords:** accelerometry, algorithm, data processing, physical activity pattern, wearable sensors

## Abstract

Physical activity (PA) is a complex human behavior, which implies that multiple dimensions need to be taken into account in order to reveal a complete picture of the PA behavior profile of an individual. This scoping review aimed to map advanced analytical methods and their summary variables, hereinafter referred to as wearable‐specific indicators of PA behavior (WIPAB), used to assess PA behavior. The strengths and limitations of those indicators as well as potential associations with certain health‐related factors were also investigated. Three databases (MEDLINE, Embase, and Web of Science) were screened for articles published in English between January 2010 and April 2020. Articles, which assessed the PA behavior, gathered objective measures of PA using tri‐axial accelerometers, and investigated WIPAB, were selected. All studies reporting WIPAB in the context of PA monitoring were synthesized and presented in four summary tables: study characteristics, details of the WIPAB, strengths, and limitations, and measures of association between those indicators and health‐related factors. In total, 7247 records were identified, of which 24 articles were included after assessing titles, abstracts, and full texts. Thirteen WIPAB were identified, which can be classified into three different categories specifically focusing on (1) the activity intensity distribution, (2) activity accumulation, and (3) the temporal correlation and regularity of the acceleration signal. Only five of the thirteen WIPAB identified in this review have been used in the literature so far to investigate the relationship between PA behavior and health, while they may provide useful additional information to the conventional PA variables.

## 1 INTRODUCTION

Less sedentary behavior and more physical activity (PA) provide important health benefits and help mitigate health risks.^1^ PA is a complex human behavior, which implies that multiple dimensions (e.g., the type, intensity, and duration) of PA need to be taken into account in order to reveal a complete picture of the PA behavior of an individual. The FITT framework (F = frequency, I = intensity, T = time, and T = type), developed by the American College of Sports Medicine, already referred to the multiple dimensions of PA and highlighted that each of those is needed to provide specific recommendations for certain health conditions.^2^ However, this was developed with self‐report methods in mind, by which further features going beyond the classical average, count and volume measures of PA are missed (e.g., regularity, temporal correlations). Indeed, summary variables such as for example the energy expenditure or the time spent sitting are only covering the intensity and/or the duration dimensions.

Other features of the PA behavior may provide relevant information. For instance, how an individual accumulates sedentary or active time may be an important complementary information to the duration itself.^3^ A complex variability, characterized by long‐range (fractal) correlations, has also been proven substantial for many physiological systems (e.g., human heartbeat or motor control).^4^, ^5^, ^6^, ^7^ This complex and multiscale temporal organization can, for instance, be altered by aging or pathological conditions (e.g., dementia, mood disorders).^4^, ^5^, ^6^, ^7^ Therefore, the complexity of the PA behavior may also provide important complementary information to the conventional variables. In fact, it was suggested that each type of PA behavior pattern may have different health implications.^8^ Consequently, the question arises whether those conventional variables are sufficient to describe the complexity of the PA behavior and to investigate associations with different health conditions. Conventional variables that follow the FITT framework may not be sensitive enough to detect between‐group differences or changes in PA,^9^ as they only reflect a part of the reality, by covering just one single dimension of PA such as for instance the duration or frequency of a specific activity. Therefore, conventional variables might suffer from a lack of precision in terms of specificity and discriminative ability. Thus, advanced analytical methods are needed in order to identify and investigate activity profiles that have so far been missed and to subsequently gain better insights into the role of PA behavior in health, which is a prerequisite for designing effective interventions.

Wearable devices have already proven valuable to monitor PA in free‐living conditions,^10^ but technological advances may enable even more progress. The access to the raw signal (i.e., time series) of the wearable device presents the opportunity to extract many features of the signal and the PA behavior, but it also confronts the researcher with a new responsibility: the post‐processing and analysis of a large amount of data toward time aggregated descriptors of PA behavior. Designing the post‐processing requires knowledge in biomechanics, physiology, mathematics, engineering, computer science, and statistics.^11^ In addition, the more complex the data processing and the final metric, the more difficult the interpretation and the translation of the metric into recommendations. This might be two of the reasons why conventional summary variables, such as time spent in specific PA intensities (e.g., moderate to vigorous PA), which are easy to calculate are often used,^12^ even though more features of the acceleration signal could be employed.

Recently, *wearable*‐*specific indicators of PA behavior* (WIPAB), defined as “advanced analytical methods and their corresponding summary variables assessing the PA behavior from wearable sensor time series data beyond the FITT framework,” have been proposed for the assessment of PA.^13^, ^14^, ^15^ These WIPAB, which are exclusive to wearables as they cannot be derived from self‐reported methods, aim to capture the complex nature of the PA behavior. However, as the use of WIPAB is still quite new in health research, no systematic review has covered the domain of WIPAB so far. Therefore, the main purpose of this scoping review is to map the wearable‐specific indicators that can be computed from tri‐axial accelerometers and used to provide an all‐encompassing assessment of the PA behavior of an individual in order to provide a systematic summary of available measures.^16^ In this review, we focus on WIPAB derived from tri‐axial accelerometer data only (raw and summary‐level data) due to their widespread use in research. The secondary aims consist in identifying the main strengths and limitations of these WIPAB and to identify those that have been used to study the association between PA and certain health conditions.

## 2 METHODS

### 2.1 Protocol and registration

The protocol has been registered with the Open Science Framework (OSF, https://osf.io/yxgmb) and previously published elsewhere.^16^ This scoping review was conducted according to the Preferred Reporting Items for Systematic Reviews and Meta‐Analyses Extension for Scoping Reviews (PRISMA‐ScR) guidelines.^17^

### 2.2 Eligibility criteria

The population, concept, and context (PCC) framework, recommended by the Joanna Briggs Institute for Scoping Reviews,^18^ was used to define the inclusion and exclusion criteria. The targeted population is humans without restriction based on age, sex, or health condition. The concept is characterized by the outcome (PA behavior), the device (accelerometer), and the methods (WIPAB). The context is defined by daily life PA behavior quantified in free‐living conditions. Besides, a conceptual framework,^16^ which presents a non‐exhaustive list of already available WIPAB, was developed by the research team to illustrate the motivation for the present review and to define the area of interest.

Thus, articles were included if they (1) assessed PA behavior, (2) gathered objective measures of PA using research or commercial grade tri‐axial accelerometers, (3) used advanced analytical methods based on time series techniques, and (4) calculated the corresponding multidimensional PA summary variables based on accelerometer data. The selection of articles was limited to studies published in English, including articles in press, from January 2010 to April 2020 (date of the last iteration of literature search). This was motivated by the fact that this review focused on WIPAB, which surpass the information provided by conventional summary variables (e.g., the time spent at a certain intensity) often used before 2010.^11^, ^19^ Studies including non‐human subjects, gray literature, reviews, and opinion articles were excluded. However, reference lists from the reviews on related topics were screened for additional potentially relevant articles. Studies investigating methods that combine data from accelerometers and other sensors as, for example, thermometers, inclinometers, pulsometers, light sensors, GPS, or skin conductance sensors were also excluded. Studies identifying the type of PA through classification algorithms (e.g., machine learning methods) were excluded because the output of these studies does not provide any information on other dimensions than PA type. Also, some systematic reviews were conducted on this topic in recent years.^20^, ^21^ Studies investigating sleep or sedentary behavior as a single standalone sub‐domain of the physical behavior were also excluded as we aimed to target all‐encompassing assessments of the PA behavior. Studies on circadian rhythm analyses were excluded because the indicators mainly only cover the timing and volume domains, while systematic reviews were already conducted on this topic in recent years.^22^, ^23^ Studies focusing on population‐based analysis, and thus, on the classification of an individual into different PA profiles (e.g., cluster analysis, latent profile analysis, and functional principal component analysis) were excluded, as they do not provide a single summary variable for each individual. Similarly, statistical analyses based on conventional variables (e.g., isotemporal substitution analysis, and machine learning) were also excluded as they represent the next step after the identification of wearable‐specific indicators of PA behavior. The selection of the most appropriate statistical approach is, however, as crucial as the selection of the most suitable indicator. A recent consensus statement provides a helpful overview of the mathematical transformations and statistical analysis.^24^

### 2.3 Search strategy

Three databases were searched to find potentially relevant literature, including MEDLINE (PubMed), Embase, and Web of Science. Based on the PCC framework, a search strategy was developed by means of an extensive list of synonyms for PA, the devices used and the outcome analyzed, using keywords from important publications related to this topic. The search strategy was developed in cooperation with the academic librarian of our institution. To identify relevant literature, the search was restricted to the “title, abstract, and keywords” fields. Emtree (Embase) and MeSh (MEDLINE) terms were not used to develop the search equation, because some of the important keywords were expanding into terms that were not relevant to our research aim. Web of Science does not have a comparable subject heading tool (thesauri). The search strategy was agreed on by TG, AB, and LM, and the search was conducted by TG and AB. The search strategy for the Embase database can be found in the table in Appendix S1.

### 2.4 Data management

Articles retrieved from the electronic databases were downloaded into EndNote^®^ (version 8). Endnote was used for the removal of duplicates and the sorting of the articles based on the inclusion and exclusion criteria. Furthermore, endnote was used to manage full texts of the relevant literature to be included.

### 2.5 Selection process

The records retrieved from the abovementioned databases were combined, and all the duplicates were removed. A two‐step process was adopted to identify relevant articles. In the first step, two authors (TG and AB) screened the titles and abstracts of the records and those which did not meet the eligibility criteria were excluded. The results were compared to ensure consistency and resolve any incongruity. During this step, the reference list from relevant reviews as well as potential studies that may have resulted from relevant protocols was also searched.

During the second step, two authors (TG and AB) screened the full texts of the selected articles for eligibility. In case of non‐eligibility, the reason for exclusion was recorded. In addition, the reference list of each included study was screened for further relevant articles. The articles meeting all the inclusion criteria were kept for data extraction. Disagreements regarding the inclusion or exclusion of a specific article were discussed in a team meeting, and discrepancies were resolved by consulting a third author (LM) when necessary.

### 2.6 Data extraction process

In order to describe the different WIPAB, their interpretation, strengths, and limitations as well as their association with different health conditions, the data from the finally selected articles were extracted by two authors (TG and AB) separately by means of a data extraction form. The data extraction form was tested on five different studies to ensure their functionality. The extracted data were compared regularly to ensure consistency. The data extracted from each of the included articles relates to the following key information, providing the structure for the reported results. First, the study characteristics are presented, including the author(s), the year of publication, the study design, details on the study population (sample size, description of the population, sex, mean age, and age range), the device used, the wear location, the length of the follow‐up period (measurement length), details on the periods analyzed and on the definition of valid data. Secondly, the identified WIPAB are described in detail, including the name of the indicator, a description, and an interpretation. An overview of the software used to determine the WIPAB can be found in the table in Appendix S2. Thirdly, the strengths and limitations of the identified WIPAB are pointed out, which are both based on those reported in the identified sources as well as on our expertise and critical evaluation. Lastly, the associations between the identified WIPAB and health‐related factors are presented, including the name of the indicator, the population analyzed, the health‐related factor, a specification on the analysis (statistical model and adjustment for potential confounders) used, and the outcome of the study (associations and their direction).

## 3 RESULTS

**FIGURE 1:**
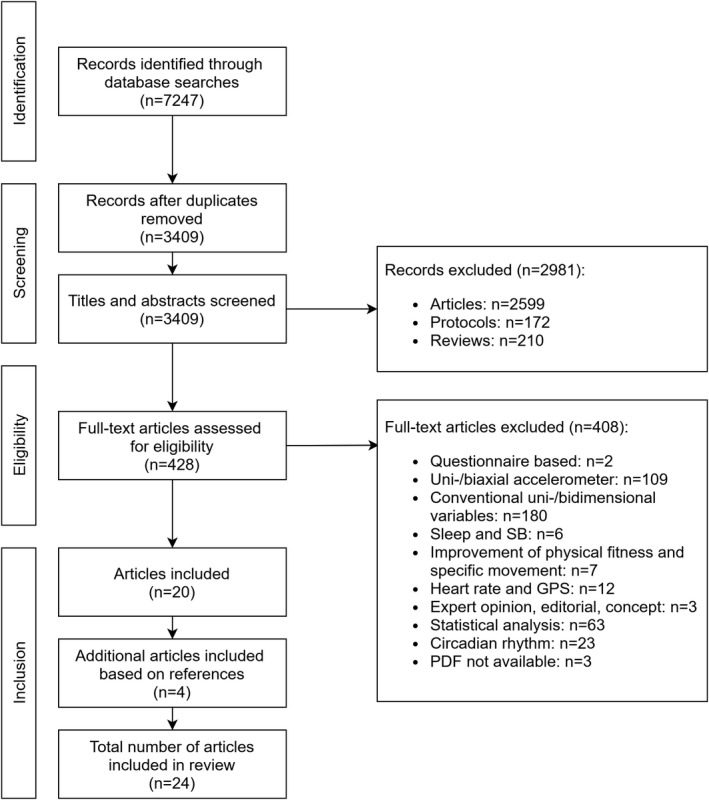
Flow‐chart of the article selection

The selection process is presented in the flow diagram (Figure 1). In total, 7247 records were identified through the database searches. Overall, 3838 duplicates were removed, resulting in 3409 records, which were included in the title and abstract screening process. After having removed 2981 records due to non‐fulfillment of the eligibility criteria, 428 full‐text articles were screened. Twenty articles fulfilled the eligibility criteria. After screening the reference lists of those 20 articles, another four articles were included, resulting in 24 articles included in the final review. Additionally, fourteen reviews and six protocols seemed to be of potential relevance and were checked in detail (see Section 2). However, none of the studies related to the protocols and none of the articles from the reviews’ reference lists were additionally included.

### 3.1 Study characteristics

A detailed description of the included studies is presented in Table 1. Four studies were performed in children and adolescents (<18 years),^9^, ^13^, ^25^, ^26^ five studies in adults younger than 65,^3^, ^27^, ^28^, ^29^, ^30^ six studies in elderly older than 65 years^4^, ^6^, ^14^, ^15^, ^31^, ^32^ and four studies investigated both adults and elderly.^33^, ^34^, ^35^, ^36^ Five studies included age groups from children to elderly.^37^, ^38^, ^39^, ^40^, ^41^

**TABLE 1:** Details of the included studies

First author (year)	Study design	Population				Device used	Wear location	Follow‐up period/period analyzed	Valid data definition
		Sample size	Population description	Sex (female/male)	Mean age (SD), range				
Barry et al. (2015)	Observational study	97	Community‐dwelling older adults	47/50	69.2 (7.7)	activPAL	Upper thigh	7 days/full monitoring period	N/A
Buchan et al. (2019)	Cross‐sectional study	246	Children	138/108	9.6 (1.4)	AG wGT3X‐BT	Non‐dominant wrist	7 days/full monitoring period	≥16 h/day for at least 3 days
Chastin et al. (2010)	Cross‐sectional study	126	Healthy subjects with an active occupation (*n* = 53)	5/48	39.2, 23–59	activPAL	Upper thigh	3–7 days/full monitoring period	N/A
			Healthy subjects with a sedentary occupation (*n* = 54)	10/44	39.9, 22–60				
			Subjects with low back pain (*n* = 5)	3/2	45.0, 40–51				
			Subjects with chronic fatigue syndrome (*n* = 14)	11/3	48.3, 34–63				
Chen et al. (2015)	Cross‐sectional study	111	Lung cancer patients	58/53	64.28 (10.86), 37–88	MicroMini ActiGraph	Non‐dominant wrist	3 days/full monitoring period	Complete data
Dunton et al. (2019)	Longitudinal observational study	169	Children	92/77	10.16 (0.87), 8–12	AG GT3X	Waist	6 × 7 days/full monitoring period	≥10 h/day for at least 1 day during at least 2 of the 6 assessments
Fairclough et al. (2020)	Secondary data analysis from a cross‐sectional observation study (phase 1) and interventional study (phase 3)	296	Children	156/140	10.0 (0.4), 9–10	AG GT9X	Non‐dominant wrist	7 days/school hours (school start to end)	100% wear time during school hours for at least 3 days
Fairclough et al. (2019)	Secondary data analysis from an interventional study	145	Children	83/62	9.6 (0.3), 9–10	AG GT9X	Non‐dominant wrist	7 days/full monitoring period	≥16 h/day for at least 3 days
Fortune et al. (2017)	Cross‐sectional study	11	Older women	11/0	77.0 (9.0)	Custom‐built activity monitors	Waist and ankle	4 days/full monitoring period	≥10 h/day
Hauge et al. (2011)	Case‐control study	81	Chronic psychotic patients (*n* = 24)	3/21	47.4 (11.1), 27–69	AW (CamNtech)	Right wrist	14 days/full monitoring period and continuous 5 h (300 min)	N/A
			Mood disorders (*n* = 25)	11/14	42.9 (10.7)				
			Healthy controls (*n* = 32)	20/12	38.2 (13.0), 21–66				
Hu et al. (2016)	Longitudinal randomized control trial	189	Residents in assisted care facilities with diagnosed dementia	170/19	85.7 (5.6), 70–96	AW (CamNtech)	Non‐dominant wrist	7–14 (baseline, after 6 weeks of treatment onset, and subsequently every 6 months after treatment onsets)/12 h during daytime	N/A
Keadle et al. (2017)	Study 1: randomized‐crossover study	10	Recreationally active subjects	6/4	25.2 (5.7)	activPAL	Right thigh	2 × 7 days/waking hours	N/A
	Study 2: randomized‐crossover study	10	Regular active subjects	N/A	25.5 (4.8)	activPAL	Thigh	N/A	N/A
	Study 3: four‐arm randomized trial	58	Overweight and obese subjects, non‐exercisers	39/19	43.2 (5.4)	activPAL	Thigh	3 × 7 days/waking hours	N/A
	Study 4: observational study	422	Community‐dwelling sample	222/200	39.1, 12–75	activPAL	Thigh	N/A	N/A
Krane‐Gartiser et al. (2018)	Case series	3	Subjects with bipolar disorders	N/A	Case 1: ≤30; Case 2: 55; Case 3: between 60 and 65	AW‐Spec (PRes)	Wrist	First few days of admission/24 h for each admission	At least 2 recordings from separate hospital admissions
Krane‐Gartiser et al. (2014)	Case‐control study	58	Acutely hospitalized inpatients with mania (*n* = 18)	11/7	51.2 (15.4)	AW‐Spec (PRes)	Wrist	1 day/64‐min active morning and 64‐min active evening period	At least 64‐min of continuous motor activity in the morning and in the evening
			Bipolar depression inpatients (*n* = 12)	7/5	39.9 (15.6)				
			Healthy controls (*n* = 28)	13/15	41.7 (11.6)				
Li et al. (2018)	Longitudinal, community‐based cohort study	1097	1097 subjects with no dementia at baseline, including 855 non‐mild cognitive impairment (MCI)	844/253	Non‐Alzheimer's dementia: 81.0 (7.4); Non‐MCI: 80.1 (7.2)	Actical	Non‐dominant wrist	10 days/full monitoring period	N/A
Merilahti et al. (2016)	Cross‐sectional study	36	Residents in assisted living facilities and nursing homes without dementia	29/7	80.4 (9.0)	Vivago WristCare AM	Wrist	7–14 days/full monitoring period	At least 7 days
Pan et al. (2013)	Longitudinal observational study	88	Outpatients with idiopathic Parkinson's Disease (*n* = 56) and healthy controls (*n* = 32)	24/32 (PD patients)	61.2 (7.9) (PD patients)	MicroMini Motionlogger AG	Dominant wrist	9 × 7 days (4‐month intervals over 3 years)/waking hours	N/A
Paraschiv‐Ionescu et al. (2018)	Observational study	40	Community‐dwelling older adults	26/14	74 (6), 65–86	Physilog	Chest, mid‐sternum level	2 days/full monitoring period	Complete data
Rowlands, Dawkins et al. (2019)	Secondary data analysis	2461	Children (*n* = 145)	83/62	9.6 (0.3), 9–10	AG GT9X	Non‐dominant wrist	7 days/full monitoring period	>16 h/day for at least 3 days
			Adolescent girls (*n* = 1669)	1669/0	12.8 (0.8), 11–14	GENEActiv	Non‐dominant wrist		
			Adult office workers (*n* = 114)	91/23	41.2 (10. 9)	AG GT9X	Non‐dominant wrist		
			Adults with type 2 diabetes (*n* = 475)	171/304	64.2 (8.7), 18–75	GENEActiv	Non‐dominant wrist		
			Children (*n* = 58)	27/31	10.7 (0.8), 10–12	AG GT3X+, GENEActiv	Right hip and non‐dominant wrist	7 days/full monitoring period	>10 h/day for at least 1 day
Rowlands et al. (2018)	Cross‐sectional study	1964	Adolescent girls (*n* = 1669)	1669/0	12.8 (0.8)	GENEActiv	Non‐dominant wrist	7 days/full monitoring period	≥16 h/day for at least 3 days
			Adults with type 2 diabetes (*n* = 295)	117/178	63.2 (9.7)				
Rowlands, Fairclough et al. (2019) and Rowlands, Sherar et al. (2019)	Secondary data analysis	4937	Children (*n* = 145)	83/62	9.6 (0.3), 9–10	AG GT9X	Non‐dominant wrist	7 days/full monitoring period	>16 h/day for at least 3 days
			Adolescent girls (*n* = 1669)	1669/0	12.8 (0.8)	GENEActiv	Non‐dominant wrist		
			Adult office workers (*n* = 114)	91/23	41.2 (10.9)	AG GT9X	Non‐dominant wrist		
			Premenopausal women (*n* = 1218)	1218/0	46.2 (3.9)	Axivity AX3	Dominant wrist		
			Postmenopausal women (*n* = 1316)	1316/0	59.0 (5.1)	Axivity AX3	Dominant wrist		
			Adults with type 2 diabetes (*n* = 475)	171/304	64.2 (8.7)	GENEActiv	Non‐dominant wrist		
Scott et al. (2017)	Pilot cross‐sectional study	34	Acute episode of mania (*n* = 16)	9/7	51.22 (CI: 43.58–58.86)	AW‐Spec (PRes)	Wrist	1 day/64‐min active morning and 64‐min active evening period	At least 64‐min of continuous motor activity in the morning and in the evening
			Bipolar depression (*n* = 12)	7/5	39.92 (CI: 29.99–49.84)				
			Mixed state (*n* = 6)	3/3	42.00 (CI: 26.65–57.35)				
Taibi et al. (2013)	Pilot cross‐sectional study	8	Subjects living with HIV	4/4	48, 39–53	AW−2 (PRes)	Non‐dominant wrist	7 days/full monitoring period	N/A
Zhang et al. (2018)	Pilot intervention study	21	Younger older adults	N/A	60–70	DynaPort MoveMonitor	Lower back at L5	2 × 7 days/full monitoring period	≥16 h/day for at least 3 days in both weeks

Abbreviations: AG, ActiGraph; AM, activity monitor; AW, Actiwatch; CamNtech, Cambridge Neurotechnology; CI, confidence interval; N/A, information not available; PRes, Philips Respironics Inc.

The majority were cross‐sectional studies (*n* = 8)^3^, ^13^, ^29^, ^30^, ^31^, ^32^, ^34^, ^39^ and secondary data analysis of cross‐sectional and interventional studies (*n* = 5).^9^, ^26^, ^38^, ^40^, ^41^ Four studies investigated longitudinal data,^4^, ^6^, ^25^, ^36^ two studies had a case‐control design,^28^, ^35^ two an observational design,^14^, ^33^ one an interventional design^15^ and one paper was based on a case series.^27^ The study by Keadle et al.^37^ includes participants from four previously published studies, including two randomized crossover studies, one four‐arm randomized trial and one observational study.

The devices from ActiGraph were the most frequently used (*n* = 7),^9^, ^13^, ^25^, ^26^, ^38^, ^40^, ^41^ followed by the devices from Philips Respironics Inc. (*n* = 5),^6^, ^27^, ^28^, ^29^, ^30^ ActivInsights (*n* = 4),^38^, ^39^, ^40^, ^41^ and PAL Technologies (*n* = 3).^3^, ^33^, ^37^ The devices from CamNtech,^4^, ^35^ Ambulatory Monitoring,^34^, ^36^ and Axivity^40^, ^41^ were used in two studies each. Further brands, which were used in one study each, were McRoberts,^15^ Vivago,^32^ and Gait Up.^14^ Only one study used a custom‐built activity monitor.^31^ Furthermore, three studies used multiple devices for their investigations.^38^, ^40^, ^41^

Most of the studies attached the accelerometer on the non‐dominant wrist (*n* = 11),^4^, ^6^, ^9^, ^13^, ^26^, ^30^, ^34^, ^38^, ^39^, ^40^, ^41^ followed by the wrist (side not specified, *n* = 4)^27^, ^28^, ^29^, ^32^ as well as the thigh (*n* = 3)^3^, ^33^, ^37^ and the dominant wrist (*n* = 3).^36^, ^40^, ^41^ Further wear locations were the waist (*n* = 2),^25^, ^31^ the right hip (*n* = 1),^38^ the right wrist (*n* = 1),^35^ the ankle (*n* = 1),^31^ the lower back (height of L5, *n* = 1),^15^ and the chest (mid‐sternum level, *n* = 1).^14^ Four papers investigated two different wear locations in their studies.^31^, ^38^, ^40^, ^41^

### 3.2 Wearable‐specific indicators of PA behavior (WIPAB)

**FIGURE 2:**
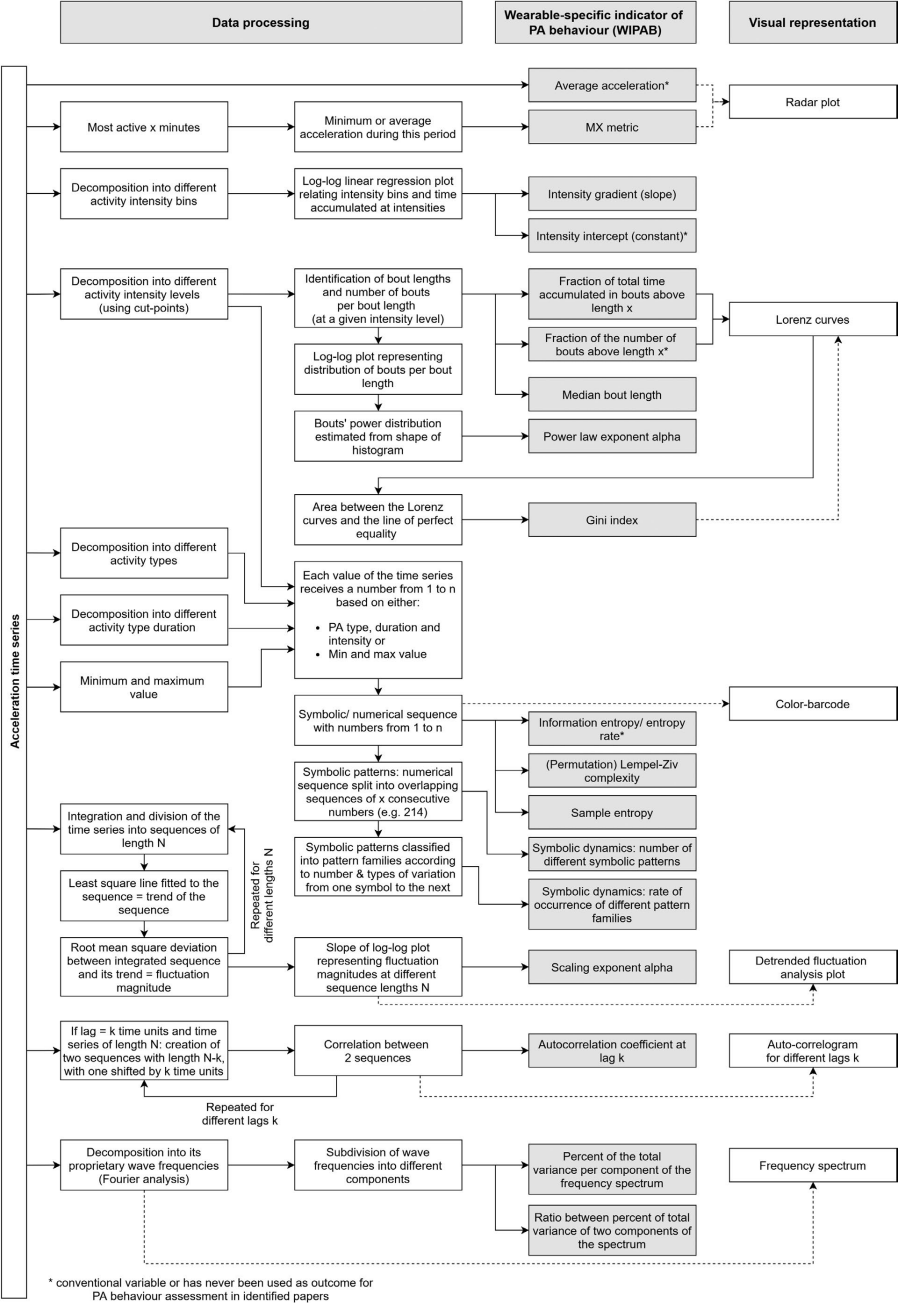
Overview of the identified wearable‐specific indicators of physical activity behavior (WIPAB)

In total, thirteen WIPAB were identified upon data extraction of the 24 included articles. Those indicators can be classified into three main categories: activity intensity distribution, activity accumulation, and temporal correlation and regularity. Detailed descriptions and interpretations of the WIPAB as well as their strengths and limitations are presented in Table 2 and Table 3, respectively. A detailed overview of the identified WIPAB and the corresponding preceding data processing steps can be found in Figure 2.

**TABLE 2:** Details of the wearable‐specific indicators of physical activity behavior (WIPAB)

WIPAB	Description	Interpretation	References
Activity intensity distribution			
Intensity gradient	The intensity gradient reflects the distribution of activity intensities across 24 h by describing the negative curvilinear relationship between activity intensities and the time accumulated at these intensities. It represents the negative slope of the double‐logarithmic plot relating intensity and time. As the time accumulated drops as intensity increases, the intensity gradient is always negative. The constant (intercept) of the linear regression equation and the *R* squared, indicating the goodness of fit of the linear model, can also be used for the description of the activity intensity distribution	A higher constant and a more negative (lower) gradient indicate a steeper drop as intensity increases, reflecting little time accumulated at midrange and higher intensities. Conversely, a lower constant and a less negative (higher) gradient indicate a shallower drop as intensity increases, reflecting more accumulated time spread across the entire intensity range	Buchan et al.^13^ Fairclough et al.^9^ Fairclough et al.^26^ Rowlands, Dawkins et al.^38^ Rowlands et al.^39^ Rowlands, Fairclough et al.^40^ Rowlands, Sherar et al.^41^
MX metric	The MX metric represents the minimum or average acceleration value above which a person's most active *x* minutes are accumulated, for example, most active 30 or 60 min. Using a range of different MX metrics (translational metrics) illustrates the 24‐h activity profile, complementarily to the intensity gradient and average acceleration (analytical metrics). The MX metric can be plotted on a radar chart to illustrate the activity profile	A higher MX metric indicates a more intense physical activity behavior for a defined time period of, for example, 30 min	Fairclough et al.^26^ Rowlands, Dawkins et al.^38^ Rowlands, Fairclough et al.^40^ Rowlands, Sherar et al.^41^
Activity accumulation			
Power‐law exponent alpha	The power‐law exponent alpha is a measure that describes the distribution of bouts according to their duration for a given activity intensity. The relationship between bout length and density of bouts is plotted on a logarithmic scale. The power distribution of the bouts, estimated from the shape of the histogram, is characterized by the power‐law exponent alpha	A lower power‐law exponent alpha indicates an accumulation pattern with a greater proportion of longer bouts. A larger power‐law exponent alpha indicates an accumulation pattern with a greater proportion of shorter bouts at a specific intensity	Barry et al.^33^ Chastin and Granat^3^ Fortune et al.^31^ Keadle et al.^37^
Median bout length	The median bout length (*x*_1/2_) gives some indication of a subject (or population) preferred bout length for a given activity intensity and is directly related to the power‐law exponent alpha	The higher the median bout length, the longer the favored duration spent at a specific activity intensity	Chastin and Granat^3^ Fortune et al.^31^
Proportion of total time accumulated in bouts longer than *x*	The proportion (fraction) of total time accumulated in bouts longer than a certain length *x* (*W_x_*). The replacement of *x* by *x*_1/2_ would give the proportion of total time accumulated in bouts longer than the median bout length Plotting *W_x_* against the proportion of the number of bouts above a certain length *x* gives the Lorenz curves, which is used to calculate the Gini index	Higher values indicate a greater imbalance between the number of bouts and their contribution to accumulation of time at a specific activity intensity	Chastin and Granat^3^ Fortune et al.^31^ Keadle et al.^37^
Gini index	The Gini index is a standardized measure of dispersion. It represents the variability in bout lengths for a given activity intensity. First, the cumulative proportion of time at the given PA level is plotted against the cumulative proportion of the number of bouts at a given PA level above a certain length *x*, which gives the Lorenz curve. The Gini index is then calculated as the area that lies between the Lorenz curve and the line of perfect equality	The Gini index ranges from 0 to 1. Values close to 0 indicate a more even and dispersed accumulation across the bout lengths during the monitoring period. Conversely, values close to 1 indicate a largely unequal physical activity distribution; thus, the activity bouts are highly unequal in length. The larger the inequality of bout lengths is, the higher becomes the Gini index and the larger is the area under the Lorenz curve^36^	Chastin and Granat^3^ Dunton et al.^25^ Fortune et al.^31^ Keadle et al.^37^
Temporal correlation and regularity			
Scaling exponent alpha	Detrended fluctuations analysis (DFA) can be used to determine long‐range temporal correlations (self‐similarity) in the activity fluctuations. Therefore, a time series is integrated and then divided into boxes of equal length. A least square line, representing the trend in that box, is fitted to each box. The magnitude of the fluctuations is then calculated based on the root‐mean‐square deviation between the integrated time series and its trend in each box. This computation is repeated over different box sizes (time scales). Finally, the magnitude of the fluctuations is plotted against the box sizes (time scales) in a double‐logarithmic plot. The slope of this straight line determines the fractal scaling exponent alpha, which provides a measure of the correlation property in the signal^51^	Scaling exponent alpha values lower than 0.5 indicate negative correlations; thus, larger values are more likely to be followed by small values and vice versa. A value of 0.5 indicates that there are no correlations in the fluctuations (“white noise”). Values above 0.5 indicate a positive correlation in the temporal structure. Thus, large activity values are more likely to be followed by large activity values or small activity values are more likely to be followed by small activity values, respectively. An alpha value around 1 indicates the highest temporal correlation in the activity fluctuations	Hu et al.^4^ Li et al.^6^ Pan et al.^36^
Autocorrelation coefficient at lag *k*	An autocorrelation function is a mathematical measure that refers to the degree of relationship between observations that are *k* lags apart. Thus, the correlation of a time series with its own past and future values is determined. For *k* equal to 1 min (autocorrelation at lag 1 min), the correlation of this time series with itself lagged 1 min is investigated. The 24‐h autocorrelation (*k* equal to 1440 min) quantifies the regularity and consistency of activity patterns between days, thus time series that are 24 h (ie, 1440 min) apart. Therefore, the activity levels from each minute of clock time are compared across successive days (ie, the activity level at 08:00 AM on a given day is compared to the activity level at 08:00 AM on the next day)	The autocorrelation coefficient ranges from −1 to 1. Coefficients closer to 1 indicate a stronger correlation, thus perfectly matching data. For the 24‐h autocorrelation, a higher coefficient indicates a more robust circadian rhythm. Conversely, coefficients closer to −1 indicate an exact opposite of the daily activity timing between days. Coefficients closer to 0 indicate a weaker correlation, thus a large day‐to‐day variation in the activity patterns	Krane‐Gartiser et al.^28^ Scott et al.^29^ Chen et al.^34^ Merilahti and Korhonen^32^ Taibi et al.^30^
Fourier analysis	The Fourier analysis can be used to decompose time series data into its proprietary wave frequencies that make up the signal. In order to improve the frequency resolution and algorithm efficiency, sequence lengths that are potencies of 2 (e.g., 32, 64, and 128 min or h) are preferred. In the context of PA pattern analysis, the Fourier analysis was used to subdivide activity patterns into patterns that repeated itself with a high frequency (e.g., 0.0021–0.0083 Hz, corresponding to a period of repetition from 2 to 8 min) or a low frequency (e.g., 0.00026–0.0021 Hz, corresponding to a period of repetition from 8 to 64 min). The results can either be presented as percent of the total variance per component of the spectrum analyzed (e.g., only period from 2 to 8 min) or as ratio between the percent of the total variance of two components of the spectrum (e.g., low‐frequency part compared to the high‐frequency part)	If the result is referring to a single component of the spectrum, a higher value indicates a higher contribution to the total variance in the corresponding spectrum. If analyzed as a ratio between two different components of the spectrum, a higher value indicates, for example, a higher contribution to the total variance of the high‐frequency part as compared to the low‐frequency part of the spectrum or vice versa	Hauge et al.^35^ Krane‐Gartiser et al.^28^ Scott et al.^29^
Sample entropy	The sample entropy is a nonlinear measure that quantifies the degree of regularity (complexity) of a time series by analyzing the presence of similar sub‐patterns in the data sequence. Sample entropy is the negative value of the natural logarithm of the conditional probability that two similar sequences of *m* points, that match point‐wise within a tolerance interval, remain similar at the next point *m* + 1, counting each vector over all other vectors except on itself^41^	A high value of sample entropy indicates an increased disorder, thus a time series with a high complexity, irregularity, and unpredictability. Conversely, a low value indicates a more regular time series	Hauge et al.^35^ Krane‐Gartiser et al.^28^ Krane‐Gartiser et al.^27^ Scott et al.^29^
Lempel‐Ziv complexity	The Lempel‐Ziv complexity is a structural‐dynamic and non‐parametric complexity measure that captures the diversity of states and the dynamics of change between states. Thus, the approach prior requires the reduction of raw accelerometry data in PA states (e.g., based on intensity, duration, and type of PA). It quantifies the number of distinct temporal sub‐sequences of physical activity states and the rate of their recurrence	A higher Lempel‐Ziv complexity value indicates a greater chance of the occurrence of new sub‐sequences and thus a more complex temporal/dynamical behavior^37^	Paraschiv‐Ionescu et al.^14^ Zhang et al.^15^
Permutation Lempel‐Ziv complexity	The permutation Lempel‐Ziv complexity is an improved version of the classical Lempel‐Ziv complexity, aiming to increase the performance regarding sensitivity for complexity assessment and robustness to potential signal artifacts. Compared to the classical Lempel‐Ziv complexity measure, it only considers the order relations between the values in the sequence and not the absolute values themselves^40^	A higher permutation Lempel‐Ziv complexity value indicates higher complexity in the time series data. Conversely, lower values indicate less complexity in the data	Paraschiv‐Ionescu et al.^14^
Symbolic dynamics	Symbolic dynamics is a measure of nonlinear complexity. The time series is transformed into a sequence of integers (ie, symbols) consisting of a string of numbers from 1 to *n*. Accordingly, the difference between the minimum and maximum value of the analyzed series is divided into *n* equal portions and each value of the series receives a number from 1 to *n*. The series is then divided into overlapping sequences (symbolic patterns) of three consecutive numbers All symbolic patterns, consisting of the three numbers, are then grouped without any loss into four different pattern families according to the number and types of variations from one symbol to the next: (1) A pattern with no variation, where all the symbols are equal (e.g., 333), (2) a pattern with only one variation where two consecutive symbols are equal and the remaining symbol is different (e.g., 331), (3) a pattern with two like variations, where the three symbols either ascend or descend (e.g., 641 or 235), and (4) a pattern with two unlike variations, with both ascending and descending progressions (e.g., 312 or 451). This pattern redundancy reduction strategy is motivated by the aim to group all possible patterns into four categories characterized by different frequency contents^43^	The total number of different symbolic patterns, consisting of the three numbers, already gives an indication of the variability of the time series. The rates of occurrence of the four pattern families, presented as the percentage of the total number of patterns analyzed, indicate the complexity of the time series	Krane‐Gartiser et al.^28^

**TABLE 3:** Strengths and limitations of the identified wearable‐specific indicators of physical activity behavior (WIPAB)

WIPAB	Strengths	Limitations	References
Intensity gradient	Independence from cut points Can be combined with the average acceleration to investigate independent, additive, or interactive associations of volume and intensity distribution with health Captures time spent across the intensity spectrum without having to handle compositional challenges	Only limited reference values in the literature No information on temporal accumulation Likely dependent on epoch length and acceleration metric choice Magnitude of the intensity gradient dependent on the size of intensity bins used to summarize the acceleration signal	Buchan et al.^13^ Fairclough et al.^9^ Fairclough et al.^26^ Rowlands, Dawkins et al.^38^ Rowlands et al.^39^ Rowlands, Fairclough et al.^40^ Rowlands, Sherar et al.^41^
MX metric	Independence from cut points Post‐hoc comparison with cut points	Agreement on key MX metrics needed, so a decision needs to be made on time thresholds No information on temporal accumulation	Fairclough et al.^26^ Rowlands, Dawkins et al.^38^ Rowlands, Fairclough et al.^40^ Rowlands, Sherar et al.^41^
Power‐law exponent alpha	Information on bout length distribution Identification of different PA behavior strategies (e.g., proportion of longer bout lengths in the accumulation of time spent at a specific intensity)	Difficult to interpret in terms of typical (ie, subject or population preferred) bout length. Therefore, complementary metrics such as *x*_1/2_ (median bout length) and *W*_1/2_ (fraction of total time accumulated in bouts longer than *x*_1/2_) were proposed	Barry et al.^33^ Chastin and Granat^3^ Fortune et al.^31^ Keadle et al.^37^
Median bout length	Information on the preferred bout length of a specific subject or population Is directly related to the power‐law exponent alpha	Present only limited information on the bout length distribution	Chastin and Granat^3^ Fortune et al.^31^
Proportion of total time accumulated in bouts longer than *x*	Information on accumulation pattern of a specific activity intensity Can be combined with the proportion of the number of bouts above a certain length *x* to form the Lorenz curves	Present only limited information on the bout length distribution	Chastin and Granat^3^ Fortune et al.^31^ Keadle et al.^37^
Gini index	Information on inequality in bout length distribution Non‐parametric	Only limited reference values in the literature	Chastin and Granat^3^ Dunton et al.^25^ Fortune et al.^31^ Keadle et al.^37^
Scaling exponent alpha	Information on temporal correlations	Requires that the time scales of interest occur in all recordings. No clear guidance on the selection of a suitable range of time scales Originally developed for ECG analysis with long series of heartbeats, which may make it less suitable for relatively short series of behavior bouts per day Not suitable for rare behaviors, for example, vigorous activity	Hu et al.^4^ Li et al.^6^ Pan et al.^36^
Autocorrelation coefficient at lag *k*	Information on temporal correlations	Strength of correlation potentially specific to sensor location, and signal processing steps	Autocorrelation at lag 1 min: Krane‐Gartiser et al.^28^ Scott et al.^29^ Autocorrelation at lag 24 h: Chen et al.^34^ Merilahti et al.^32^ Taibi et al.^30^
Fourier analysis	Information on the variance of different frequency spectrum components	Does not capture temporal structure Less suitable for rare behaviors, especially when these rare behaviors have a low signal magnitude	Hauge et al.^35^ Krane‐Gartiser et al.^28^ Scott et al.^29^
Sample entropy	Information of the regularity of the time series Independence from time series length Robustness regarding outliers	Resting periods can skew the results	Hauge et al.^35^ Krane‐Gartiser et al.^28^ Krane‐Gartiser et al.^27^ Scott et al.^29^
Lempel‐Ziv complexity	Information on diversity of states and dynamics of change between different states (variability of the time series) If applied to PA states: quantity and quality dimension of daily activities are taken into account	Transformation of the original signal into a finite sequence with only binary elements (coarse‐graining process) Dependency from the resolution of the time series Susceptible to noise, due to sensitivity to the amplitude distribution Sensitive to the length of the sequence	Paraschiv‐Ionescu et al.^14^ Zhang et al.^15^
Permutation Lempel‐Ziv complexity	Information on diversity of states and dynamics of change between different states (variability of the time series) If applied to PA states: quantity and quality dimension of daily activities are taken into account Use of permutations (motifs) of a chosen length (instead of a coarse‐graining process) to estimate complexity Robustness to noise as it only considers the order relations between the values in the time series	Sensitive to the length of the sequence	Paraschiv‐Ionescu et al.^14^
Symbolic dynamics	Information on the variability of the time series	Depends on prior classification of the time series in symbolic patterns	Krane‐Gartiser et al.^28^

#### 3.2.1 Activity intensity distribution

The intensity gradient^9^, ^13^, ^26^, ^38^, ^39^, ^40^, ^41^ as well as a set of the MX metrics^26^, ^38^, ^40^, ^41^ were used to investigate the PA intensity distribution across the monitoring period.

#### 3.2.2 Activity accumulation

The activity accumulation variables are measures of dispersion that describe the distribution of activity durations (bout lengths) on a time axis, independently of the intensity. The power‐law exponent alpha,^3^, ^31^, ^33^, ^37^ the median bout length,^3^, ^31^ the Gini index,^3^, ^25^, ^31^, ^37^ and the proportion of total time accumulated in bouts longer than *x* ^3^, ^31^, ^37^ were used to quantify patterns of accumulation.

#### 3.2.3 Temporal correlation and regularity

The scaling exponent alpha,^4^, ^6^, ^36^ the Fourier analysis,^28^, ^29^, ^35^ and the autocorrelation at lag 24 h^30^, ^32^, ^34^ and lag 1 min^28^, ^29^ investigate temporal correlations in the signal. Similarly, the sample entropy,^27^, ^28^, ^29^, ^35^ the Lempel‐Ziv complexity,^14^, ^15^ the permutation Lempel‐Ziv complexity,^14^ and the symbolic dynamics approach^28^ are analytical methods used to assessed the regularity or randomness of a signal.

### 3.3 Association with health conditions

Ten studies investigated potential associations between the identified WIPAB and certain health‐related factors (Table 4). The intensity gradient was investigated in four studies.^9^, ^13^, ^39^, ^40^ It was found to be negatively associated with BMI z‐score, waist‐to‐height ratio, metabolic syndrome, and percent body fat in children, as well as with BMI in adults with type 2 diabetes, and with percent body fat in adult office workers and pre‐ and postmenopausal women, even after adjustment for commonly used confounders (e.g., age and sex). In adults with type 2 diabetes, the negative association between intensity gradient and percent body fat remained significant after adjustment for potential confounders in one study^40^ and became non‐significant in another study.^39^ A positive association was found with cardiorespiratory fitness and health‐related quality of life in children, as well as with bone density T‐score in pre‐ and postmenopausal women, and with physical fitness in adults with type 2 diabetes (adjusted models). Nearly all associations remained significant after further adjustment for the conventional variable average acceleration, indicating independent associations. Only the associations between the intensity gradient and health‐related quality of life in children,^9^ percent body fat in adult office workers,^40^ bone density T‐score in premenopausal women,^40^ BMI and percent body fat in adults with type 2 diabetes^39^ were not independent of average acceleration.

**TABLE 4:** Correlations and measures of association between the wearable‐specific indicators of physical activity behavior (WIPAB) and health‐related factors

WIPAB	Reference	Population	Health‐related factor	Statistical model	Adjustment of the statistical model	Associations and their direction
Intensity gradient	Buchan et al.^13^	Children	BMI z‐score	MLRM (1)	–	↘
				MLRM (2)	1 + age, sex	↘
				MLRM (3)	2 + conventional variable (average acceleration)	↘
	Fairclough et al.^9^	Children	BMI z‐score	LME (1)	School level clustering	↘
				LME (2)	1 + sex, maturation, socio‐economic status	↘
				LME (3)	2 + conventional variable (average acceleration)	↘
			Waist‐to‐height ratio	LME (1)	School level clustering	↘
				LME (2)	1 + sex, maturation, socio‐economic status	↘
				LME (3)	2 + conventional variable (average acceleration)	↘
			Cardiorespiratory fitness	LME (1)	School level clustering	↗
				LME (2)	1 + sex, maturation, socio‐economic status	↗
				LME (3)	2 + conventional variable (average acceleration)	↗
			Metabolic syndrome score	LME (1)	School level clustering	↘
				LME (2)	1 + sex, maturation, socio‐economic status	↘
				LME (3)	2 + conventional variable (average acceleration)	↘
			Health‐related quality of life	LME (1)	School level clustering	↗
				LME (2)	1 + sex, maturation, socio‐economic status	↗
				LME (3)	2 + conventional variable (average acceleration)	–
	Rowlands, Fairclough et al.^40^	Children (9–10 years)	BMI z‐score	GLM (1)	School level clustering	↘
				GLM (2)	1 + age, sex, socio‐economic status	↘
				GLM (3)	2 + conventional variable (average acceleration)	↘
		Adolescent girls (11–12 years)	Percent body fat	GLM (1)	School level clustering	↘
				GLM (2)	1 + age, socio‐economic status, biological maturity, ethnicity	↘
				GLM (3)	2 + conventional variable (average acceleration)	↘
		Adolescent girls (13–14 years)	Percent body fat	GLM (1)	School level clustering	↘
				GLM (2)	1 + age, socio‐economic status, biological maturity, ethnicity	↘
				GLM (3)	2 + conventional variable (average acceleration)	↘
		Adult office workers	Percent body fat	MLRM (1)	–	↘
				MLRM (2)	1 + age, sex, socio‐economic status, ethnicity	↘
				MLRM (3)	2 + conventional variable (average acceleration)	–
		Premenopausal women	Percent body fat	MLRM (1)	–	↘
				MLRM (2)	1 + age	↘
				MLRM (3)	2 + conventional variable (average acceleration)	↘
			Bone density T‐score	MLRM (1)	–	↗
				MLRM (2)	1 + age, height, fat mass, fat‐free mass, alcohol consumption, age at menarche, years taking contraceptives, currently on contraceptives	↗
				MLRM (3)	2 + conventional variable (average acceleration)	–
		Postmenopausal women	Percent body fat	MLRM (1)	–	↘
				MLRM (2)	1 + age	↘
				MLRM (3)	2 + conventional variable (average acceleration)	↘
			Bone density T‐score	MLRM (1)	–	↗
				MLRM (2)	1 + age, height, fat mass, fat‐free mass, alcohol consumption, age at menarche, years taking contraceptives, years since menopause	↗
				MLRM (3)	2 + conventional variable (average acceleration)	↗
		Adults with type 2 diabetes	Percent body fat	MLRM (1)	–	↘
				MLRM (2)	1 + age, sex, socio‐economic status, ethnicity	↘
				MLRM (3)	2 + conventional variable (average acceleration)	↘
			Short Physical Performance Battery score	MLRM (1)	–	↗
				MLRM (2)	1 + age, sex, socio‐economic status, ethnicity	↗
				MLRM (3)	2 + conventional variable (average acceleration)	↗
	Rowlands et al.^39^	Adolescent girls	BMI z‐score	GLM (1)	School level clustering	↘
				GLM (2)	1 + age, biological maturity, socio‐economic status, ethnicity	↘
				GLM (3)	2 + conventional variable (average acceleration)	↘
			Percent body fat	GLM (1)	School level clustering	↘
				GLM (2)	1 + age, biological maturity, socio‐economic status, ethnicity	↘
				GLM (3)	2 + conventional variable (average acceleration)	↘
		Adults with type 2 diabetes	BMI	MLRM (1)	–	↘
				MLRM (2)	1 + age, sex, socio‐economic status, ethnicity	↘
				MLRM (3)	2 + conventional variable (average acceleration)	–
			Percent body fat	MLRM (1)	–	↘
				MLRM (2)	1 + age, sex, socio‐economic status, ethnicity	–
				MLRM (3)	2 + conventional variable (average acceleration)	–
			Average grip strength	MLRM (1)	–	↗
				MLRM (2)	1 + age, sex, socio‐economic status, ethnicity, percent body fat	↗
				MLRM (3)	2 + conventional variable (average acceleration)	↗
			Sit‐to‐stand test (60 repetitions)	MLRM (1)	–	↗
				MLRM (2)	1 + age, sex, socio‐economic status, ethnicity, percent body fat	↗
				MLRM (3)	2 + conventional variable (average acceleration)	↗
			Short Physical Performance Battery score	MLRM (1)	–	↗
				MLRM (2)	1 + age, sex, socio‐economic status, ethnicity, percent body fat	↗
				MLRM (3)	2 + conventional variable (average acceleration)	↗
Scaling exponent alpha	Hu et al.^4^ *Activity correlations (alpha) at small time scales (<1.5 h)*	Elderly with dementia	Mini‐Mental State Examination score (cognition)	LME	–	↗
			Multidimensional observation scale for elderly: social withdrawal behavior score	LME	–	↘
			Cornell Scale for Depression in Dementia score (mood)	LME	–	↘
	Li et al.^6^ *Per 1‐SD decrease in alpha*	Elderly without mild cognitive impairment at baseline	Incident mild cognitive impairment	Cox (1)	Age, sex, education	↗
				Cox (2)	1 + conventional variable (total daily activity level)	–
		Elderly without dementia at baseline	Incident Alzheimer's dementia	Cox (1)	Age, sex, education	↗
				Cox (2)	1 + conventional variable (total daily activity level)	↗
24‐h autocorrelation	Chen et al.^34^	Lung cancer patients	Total sleep time	Pearson's bivariate correlation	–	–
			Sleep efficiency	Pearson's bivariate correlation	–	–
			Sleep‐onset latency	Pearson's bivariate correlation	–	–
	Merilahti and Korhonen^32^	Elderly without dementia	Activities of daily living score	Spearman's rank‐correlation	–	–
Lempel‐Ziv complexity	Paraschiv‐Ionescu et al.^14^	Community‐dwelling older adults	Fall‐related psychological concerns (Falls Efficacy scale)	Spearman's rank‐correlation	–	↗
	Zhang et al.^15^ *Original PA time series*	Younger older adults	Community Balance and Mobility Scale score	Spearman's rank‐correlation	–	↗
	Zhang et al.^15^ *Smoothed PA time series*	Younger older adults	Community Balance and Mobility Scale score	Spearman's rank‐correlation	–	↗
Permutation Lempel‐Ziv complexity	Paraschiv‐Ionescu et al.^14^	Community‐dwelling older adults	Fall‐related psychological concerns (Falls Efficacy scale)	Spearman's rank‐correlation	–	↗
			Functional mobility status (Timed up‐and‐go test)	Spearman's rank‐correlation	–	↘

Abbreviations: (–), no association; (↗), positive association; (↘), negative association; Cox, Cox‐proportional hazard regression model; GLM, generalized linear model; LME, linear mixed effects model; MLRM, multiple linear regression model.

In the elderly, the scaling exponent alpha was found to be positively associated with cognitive function and negatively associated with the depression score and social withdrawal behavior.^4^ Moreover, a lower scaling exponent alpha was associated with greater risk of mild cognitive impairment and Alzheimer's dementia, even after adjustment for age, sex, and education. Only the latter remained significant after further adjustment for the conventional variable total daily activity level.^6^

No significant association was found between the 24‐h autocorrelation and total sleep time, sleep efficiency, and sleep‐onset latency in lung cancer patients.^34^ No significant correlation was found between the 24‐h autocorrelation and difficulties in performing activities of daily living in elderly without dementia.^32^

The Lempel‐Ziv complexity and the permutation Lempel‐Ziv complexity were positively correlated with fall‐related psychological concerns and the “Community Balance and Mobility Scale” score in older adults.^14^, ^15^ A negative correlation was found between the permutation Lempel‐Ziv complexity and the functional mobility status in older adults.^14^

Furthermore, four studies investigated correlations and associations between WIPAB and intra‐individual changes in health‐related factors in longitudinal (observational and interventional) studies (see table, Appendix S3).^4^, ^6^, ^9^, ^36^

## 4 DISCUSSION

The main aim of this scoping review was to map wearable‐specific indicators used to provide an all‐encompassing assessment of the PA behavior of an individual (WIPAB), as well as to identify those indicators that have already been used to study the association between PA and certain health‐related factors. In total, thirteen WIPAB were identified, which can be classified into three main categories: (1) activity intensity distribution, (2) activity accumulation, and (3) temporal correlation and regularity. The first category focuses on the activity intensities and their distributions. The second category is related to the activity durations (bout lengths) and their dispersion. The third category concerns the complexity of a PA behavior, that is, correlations over a certain time period as well as the detection of certain sub‐patterns and their reoccurrence. Hence, the first category can be seen as a combination of the intensity and duration (time) dimension of the FITT framework, while the second category is a logical continuation of the FITT framework investigating the temporal accumulation. The last category can be seen as a complementary extension of the FITT framework, by adding complexity as a new dimension. The association with health‐related factors has been investigated for only five of these WIPAB.

### 4.1 Wearable‐specific indicators of PA behavior (WIPAB)

#### 4.1.1 Activity intensity distribution

The intensity gradient and the MX metric are used to assess the distribution of activity intensities. Their major strength consists in their independence from cut points. Thus, they overcome the current limitations of the lack of comparability between studies due to the wide range of cut points used.^13^, ^26^ Furthermore, the intensity gradient can be combined with the average acceleration and thus provide a more complete 24‐h activity profile of an individual. Consequently, the approach allows investigating the independent, complementary or interactive associations of volume and intensity distribution with health.^38^ The MX metric can be compared to cut points post‐hoc, enabling the maintenance of the continuous nature of the variable and the comparison to any cut‐point or acceleration indicative of a standard activity. By plotting the MX metric, visual comparisons of within and between‐group differences can be made, thus allowing the generation of data‐driven norms.^38^ However, the MX metric depends on the wear location and may differ between monitor brands, which could hinder the comparability between studies. Furthermore, there is still no consensus on the key MX metrics to analyze with respect to health conditions. Hence, a decision on time thresholds (i.e., most active *x* minutes) needs to be made. Finally, as the MX metric and the intensity gradient ignore the temporal activity accumulation, a combination with PA accumulation indicators should be envisaged.^38^

#### 4.1.2 Activity accumulation

The power‐law exponent alpha and the Gini index are measures that quantify how sedentary or active time has been accumulated. The power‐law exponent alpha provides for example information on the distribution of the bout durations, which can be used to identify different PA behavior pattern (e.g., if a person tends to accumulate sedentary time with a higher proportion of longer bouts compared to shorter bouts).^3^, ^31^ As the power‐law exponent alpha is a unit‐less parameter, the interpretation might be more difficult. Therefore, Chastin et al.^3^ proposed two additional metrics: the median bout length (*x*_1/2_), which provides information on the preferred bout length for a specific subject or population, and *W*_1/2,_ which is the proportion of the total time at a specific intensity that is accumulated in bouts longer than the median bout length (*x*_1/2_). The generalization of the latter (*W_x_*, proportion of the total time at a specific intensity that is accumulated in bouts longer than *x*) further contributes to the calculation of the Gini index. By plotting *W_x_* against the proportion of the number of bouts of length *x*, we get the Lorenz curves, which are used to calculate the Gini index.^3^, ^42^ Hence, the Gini index, a non‐parametric measure, describes the inequality in bout durations. However, similar to the intensity gradient, there is also a lack of reference values for the Gini index in the literature. As already stated above, metrics describing both the activity intensity distribution and the activity accumulation are complementary.

#### 4.1.3 Temporal correlation and regularity

The scaling exponent alpha, the autocorrelation at lag *k* (e.g., lag 24 h or lag 1 min), and the Fourier analysis are measures that investigate temporal correlations (self‐similarities) between values to find repeating patterns. The sample entropy, the (permutation) Lempel‐Ziv complexity, and the symbolic dynamics approach quantify the amount of regularity in a time series. The particular feature of these metrics is that they take the chronological aspect into account. It should be noted that the term “Fourier analysis” and “symbolic dynamics approach” were kept in the present review, even though that they describe rather the method than the specific outcome metric, in order to be consistent with the cited papers as well as because they can have more than one outcome.

To determine the amount of regularity in an acceleration time series, specific pre‐processing (i.e., data reduction) techniques may be needed to convert the raw signal into a new numerical or symbolic sequence. In the context of the symbolic dynamics approach, the acceleration time series is divided into *n* equal portions based on the acceleration value range, and each value receives then a number from 1 to *n*. Another pre‐processing technique was applied before the use of the (permutation) Lempel‐Ziv complexity,^14^, ^15^ where a symbolic sequence was composed of different PA states. PA states are created from the combination of the PA type, intensity, and duration categories. This approach presents the advantage that both the quantity and quality dimensions of daily activities are taken into account, providing important information on the PA behavior.^14^, ^15^

In a subsequent step, entropy measures can be used to quantify the information embedded in the symbolic/numerical sequence. The Lempel‐Ziv complexity, for example, determines the number of distinct patterns and the rate of their reoccurrence in a given sequence.^43^, ^44^, ^45^ The detection of changes between different patterns is, however, dependent on the resolution of the time series.^15^ The higher the resolution, the more detailed the different pattern comprised in the signal can be described, but at the same time the resolution becomes more susceptible to noise.^15^ Therefore, Zhang et al.^15^ proposed a pre‐processing method to remove irrelevant noise from the signal in order to obtain representative values that reflect the dynamic of change of activity patterns. Compared to the Lempel‐Ziv complexity, the permutation Lempel‐Ziv complexity is more robust to signal artifacts, as it only considers the order relations between the values in the time series and not the absolute values themselves.^43^ A detailed description of the procedure to calculate the permutation Lempel‐Ziv complexity can be found in Bai et al.^43^

Similar to the Lempel‐Ziv complexity, the sample entropy estimates the regularity of a symbolic/numerical sequence. However, they both assess different aspects of the dynamic complexity comprised in a sequence.^46^ As the sample entropy quantifies the probability that two sequences that are similar for *m* points remain similar at the next point *m* + 1, it is a measure of how regular the consecutive sequences are generated chronologically in time.^46^ The sample entropy is mostly independent of the length of the time series; therefore, its use is suitable for even short time series.^47^ Sample entropy also presents robustness regarding outliers. On the contrary, sample entropy is very susceptible to resting periods, resulting in lower values for signals containing extended resting periods.^27^

If the symbolic/numerical sequence is further divided into overlapping sequences of *x* consecutive numbers in order to form different patterns (e.g., 214), the symbolic dynamics approach can be applied. Thereby, the different patterns are grouped into pattern families according to their number and types of variations from one symbol to the next. The complexity of the sequence is then quantified by the rate of occurrence of the different pattern families.^28^, ^48^, ^49^ A helpful illustration of the symbolic dynamics approach can be found in Guzzetti et al..^50^

### 4.2 Association with health conditions

The intensity gradient and the scaling exponent alpha were the most frequently used metrics to investigate potential associations with certain health‐related factors.

#### 4.2.1 Activity intensity distribution

The negative associations between the intensity gradient and BMI, waist‐to‐height ratio, and percent body fat indicate that a less negative (higher) gradient and therefore more time accumulated at midrange and higher intensities is related to better health indicators.^9^, ^13^, ^39^, ^40^ This is in line with previous findings, that overweight and a higher waist circumference are associated with lesser high‐intensity PA.^51^ The latter study highlights the necessity to use a compositional data analysis approach when dealing with conventional variables as, for example, the time spent at different activity levels. As the time during the day is finite, the different activity levels are co‐dependent.^24^, ^51^ The intensity gradient circumvents this problem, as the metric is a continuous variable and was shown to be relatively independent of the overall activity defined as average acceleration.^39^ This underlines the complementarity of those two metrics.

Physical fitness and health‐related quality of life are also positively associated with the intensity gradient.^9^, ^39^, ^40^ Similar findings were found in a recent study, where the authors concluded that among older adults a higher MVPA is associated with a lower distress, which in turn is associated with a higher global quality of life.^52^ Similarly, a positive association between children's in‐school‐hours of MVPA and health‐related quality of life could be demonstrated.^53^ The positive effect of especially the higher intensities of PA on physical fitness seems inconclusive in the literature. Previous reviews reported only low to moderate correlations between daily PA and physical fitness (here defined as maximum oxygen uptake) in adolescents, with no evidence that higher intensities are more closely related than lower intensities.^54^ Yet, a more recent study demonstrated that especially high‐intensity PA is positively associated with physical fitness in adolescents.^55^ These inconclusive results might be due to the fact that the time spent at MVPA only covers a very small percentage of the amount of PA conducted. By contrast, the intensity gradient covers all the intensity spectrum, allowing the acquisition of all the PA performed. More research is needed to investigate the association between the intensity gradient and health‐related factors.

#### 4.2.2 Temporal correlation and regularity

The scaling exponent alpha, a measure of the correlation property in the signal,^7^ was found to be positively associated with cognitive functions. The higher the self‐similarity in activity fluctuations, the lower the risk of suffering from mild cognitive impairment or Alzheimer's disease. The association between the scaling exponent alpha and the risk of developing Alzheimer's disease was thereby independent of demographic characteristics (age, sex, and education) and the total daily activity level.^6^ Additionally, attenuated activity correlations (lower alpha values) at small time scales (<1.5 h) are related to worse mood (higher depression score) and social withdrawal behavior.^4^ Indeed, previous studies suggested that a “normal” PA behavior of healthy subjects is characterized by scale invariance (self‐similarity), which means that the temporal structures and properties of fluctuations of PA patterns remain the same over different time scales (minutes to hours).^5^, ^56^ It has been demonstrated that aging and disorders such as Alzheimer's disease or chronic pain reduce the scale invariance of activity fluctuations over multiple time scales.^5^, ^57^ The association between activity correlations at small time scales (<1.5 h) and mood disorders seems, however, still ambiguous. Whereas one study confirmed the findings from Hu et al.,^4^ by observing lower autocorrelation at lag 1 min (comparable to the scaling exponent at small time scales) in patients with mania and in patients with depression in the active morning period,^28^ another study revealed that higher alpha values were associated with higher depression scores.^58^ These discrepancies might be due to different associations between varied disorders or stages of diseases and temporal activity correlations.^4^

Furthermore, the intensity gradient and the scaling exponent alpha have been associated with intra‐individual changes of certain health‐related factors.^4^, ^6^, ^9^, ^36^ This finding is particularly relevant as it demonstrates that these metrics are sensitive enough to detect changes in some health conditions or demonstrate the efficiency of interventions (see table, Appendix S3). Further research should investigate their sensitivity to changes in other health conditions.

Participants that engaged in at least 295 min of light PA per day were found to have a greater 24‐h autocorrelation coefficient, which in turn was found to be positively correlated with total sleep time and sleep efficiency as well as negatively correlated with sleep‐onset latency.^34^ This aspect is of high importance for cancer patients and for elderly, where sleep disturbances are a well‐known problem.^5^, ^34^ However, it was also found that a more stable 24‐h activity rhythm (24‐h autocorrelation coefficient) seemed to indicate a lower ability to perform daily activities in older adults. In other words, those who reported a higher difficulty in performing activities of daily living tended to have a more stable activity rhythm.^32^ Thus, a higher variance in activity patterns seems to be associated with a better functioning status in older adults living in nursing homes or assisted living facilities.^32^ The discrepancies in the associations between 24‐h autocorrelation coefficient and health outcomes might be due to the specific population living in nursing homes (older and more vulnerable individuals) or to the environment itself, which can disrupt stable activity rhythms and sleep‐wake patterns and which might affect individuals with poor functioning in a more pronounced manner.^32^

Finally, higher concern about falling in an elderly population (less confident group compared to fully confident group) was correlated with a lower complexity of PA patterns, that is, a narrower range of different movements and activities.^14^ Similarly, lower mobility and lower balance were correlated with a lower complexity of PA patterns.^14^ One may speculate that the fear of falling leads to a more cautious physical behavior (e.g., slower and narrower range of different movements and activities), resulting in a less complex PA pattern and therefore, as challenging movements and activities are missing, in a lower mobility and balance.^14^ Alternatively, both fear of falling and less complex PA pattern may be the consequences of decrease physical functioning, especially locomotion. Previous studies already described an age‐, disease‐, and fall‐related functional decline in physiological and movement complexity.^46^, ^59^

### 4.3 Limitations

This scoping review presents some limitations. First, due to the high amount of available articles on the broad area of accelerometry data and PA behavior, the inclusion criteria had to be restrictive. This might have caused a disregard of some potentially relevant articles. For example, the present review only investigated associations between health‐related factors and methods that are based on tri‐axial accelerometer data. Thus, we cannot comment on possible associations that were investigated using uni‐ and bi‐axial data. However, as the latter presents difficulties to accurately capture horizontal and complex movements, tri‐axial acceleration signals should be favored, especially if the aim consists in the assessment of health‐relevant behaviors by evaluating patterns of the PA behavior.^60^

Secondly, it should be noted that in a few cases, count‐based data were used in order to calculate a specific metric (e.g., scaling exponent alpha, Fourier analysis, sample entropy, and symbolic dynamics approach). Nevertheless, as counts are only one of many techniques of data aggregation, the identified methods are not limited to this type of data, but can also be applied to a raw acceleration time series using other data aggregation techniques. However, the associations between the WIPAB and the health‐related factors might be affected by the data aggregation due to the loss of data, and comparisons between studies might be difficult.

Lastly, studies focusing on population‐based analysis, providing a classification of an individual into different PA profiles, were beyond the scope of the present review. Nevertheless, the approach that consists in comparing the PA behavior of an individual with an entire population presents an interesting concept for the study of patterns of PA behavior associated with better health conditions.

### 4.4 Perspective

The identified WIPAB demonstrate that a more all‐encompassing assessment of the PA behavior of an individual using wearable devices is already possible. Those indicators should be used to gain further insights in the role of the PA behavior in health. The results of this scoping review may be of interest for sports scientists, clinical researches, epidemiologists, and consumer wearable device companies as they provide decisive information on future developments in data processing and on relevant feedback to the end‐user. Until now, only five of the thirteen WIPAB identified in this review have been used to investigate potential associations with health‐related factors. Therefore, we recommend that studies should investigate and report indicators of at least one of the three WIPAB categories, as they might provide important complementary information on PA behavior. The selection of the most appropriate indicator depends thereby on the research question and on the health‐factor that should be analyzed. However, more research is needed to investigate which features of PA behavior are relevant with regard to the health outcome of interest, and which WIPAB is capable of predicting the development of a disease, or detecting changes in health‐related factors. Moreover, a standardization of the calculation and interpretation of the different WIPAB should be envisaged. This would favor the use of the identified WIPAB in future studies. This knowledge will take personalized prevention a significant step forward.

## CONFLICT OF INTEREST

The authors declare that they have no conflict of interest.

## Supporting information

Supplementary Material

## Data Availability

Data sharing is not applicable to this article as no new data were created or analyzed in this study.
