# Supplementary material for: Advanced analytical methods to assess physical activity behavior using accelerometer time series: A scoping review
Source: Scand J Med Sci Sports. 2021 Nov 1;32(1):18–44. doi: 10.1111/sms.14085 (PMC9298329; doi:10.1111/sms.14085)
Supplement: Supplementary file 1 — Supplementary Material [file SMS-32-18-s001.docx]

**SUPPLEMENTAL DIGITAL CONTENT**

**Supplemental digital content 1.** Search strategy for the Embase database.

| - **Search strategy** |
| --- |
| - ('physical activity':ti,ab,kw OR 'physical activities':ti,ab,kw OR 'physical function':ti,ab,kw OR 'activity profile':ti,ab,kw OR 'activity profil*':ti,ab,kw OR 'activity pattern':ti,ab,kw OR 'activity patterns':ti,ab,kw OR 'activity behavior':ti,ab,kw OR 'activity behaviour':ti,ab,kw OR 'movement behavior':ti,ab,kw OR 'movement behaviour':ti,ab,kw OR 'physical behaviour':ti,ab,kw OR 'physical behavior':ti,ab,kw OR 'sedentary behavior':ti,ab,kw OR 'sedentary behaviour':ti,ab,kw OR 'sedentary time':ti,ab,kw OR 'daily function':ti,ab,kw OR 'habitual behavior':ti,ab,kw OR 'habitual behaviour':ti,ab,kw OR 'behavioral pattern':ti,ab,kw OR 'behavioural pattern':ti,ab,kw OR 'behavioral patterns':ti,ab,kw OR 'behavioural patterns':ti,ab,kw OR 'sedentary lifestyle':ti,ab,kw OR 'sedentary lifestyles':ti,ab,kw) - AND - ('acceleromet*':ti,ab,kw OR 'accelerometry':ti,ab,kw OR 'actigraphy':ti,ab,kw OR 'wearable sensor':ti,ab,kw OR 'wearable sensors':ti,ab,kw OR 'wearable device':ti,ab,kw OR 'wearable devices':ti,ab,kw OR 'activity track*':ti,ab,kw OR 'activity sensor':ti,ab,kw OR 'activity sensors':ti,ab,kw OR 'activity monitor*':ti,ab,kw OR 'smart device':ti,ab,kw OR 'smart devices':ti,ab,kw) - AND - ('raw':ti,ab,kw OR 'metric':ti,ab,kw OR 'metrics':ti,ab,kw OR 'algorithm':ti,ab,kw OR 'algorithms':ti,ab,kw OR 'variable':ti,ab,kw OR 'variables':ti,ab,kw OR 'approach':ti,ab,kw OR 'accelerometer-derived':ti,ab,kw OR 'accelerometer derived':ti,ab,kw OR 'movement profil*':ti,ab,kw OR 'physical activity outcome':ti,ab,kw OR 'physical activity outcomes':ti,ab,kw OR 'pa outcome':ti,ab,kw OR 'pa outcomes':ti,ab,kw OR 'sedentary behavio*r outcome':ti,ab,kw OR 'sedentary behavio*r outcomes':ti,ab,kw OR 'sb outcome':ti,ab,kw OR 'sb outcomes':ti,ab,kw) - AND - [english]/lim - AND - [humans]/lim - AND - [2010-2020]/py |

**Supplemental digital content 2.** Overview of the software and specific scripts used to determine the wearable-specific indicators of physical activity behaviour (WIPAB).

| **WIPAB** | **References** | **Software and specific scripts used** |
| --- | --- | --- |
| **Intensity gradient** | Buchan et al. (2019)  Fairclough et al. (2019)  Fairclough et al. (2020)  Rowlands, Dawkins et al. (2019)  Rowlands et al. (2018)  Rowlands, Fairclough et al. (2019)  Rowlands, Sherar et al. (2019) | “GGIR” open-source package in R |
| **MX metric** | Fairclough et al. (2020)  Rowlands, Dawkins et al. (2019)  Rowlands, Fairclough et al. (2019)  Rowlands, Sherar et al. (2019) | “GGIR” open-source package in R |
| **Power law exponent alpha** | Barry et al. (2015) | Custom built non available script |
|  | Chastin et al. (2010) | Custom built non available script but equations presented in paper |
|  | Fortune et al. (2017) | Custom built non available script |
|  | Keadle et al. (2017) | “PAactivPAL” open-source package in R (removed from CRAN repository in 2018) |
| **Median bout length** | Chastin et al. (2010) | Custom built non available script but equations presented in paper |
|  | Fortune et al. (2017) | Custom built non available script |
| **Proportion of total time accumulated in bouts longer than *x*** | Chastin et al. (2010) | Custom built non available script but equations presented in paper |
|  | Fortune et al. (2017) | Custom built non available script |
|  | Keadle et al. (2017) | “PAactivPAL” open-source package in R (removed from CRAN repository in 2018) |
| **Gini index** | Chastin et al. (2010) | Custom built non available script but equations presented in paper |
|  | Dunton et al. (2019) | “reldist” open-source package in R |
|  | Fortune et al. (2017) | Custom built non available script |
|  | Keadle et al. (2017) | “reldist” and “PAactivPAL” open-source packages in R  (PAactivPAL was removed from CRAN repository in 2018) |
| **Scaling exponent alpha** | Hu et al. (2016) | JMP Pro 11 (SAS Institute, Cary, NC) |
|  | Li et al. (2018) | Custom built non available script |
|  | Pan et al. (2013) | Action-W (version 2, Ambulatory Monitors Inc.) |
| **Autocorrelation coefficient at lag *k*** | Krane-Gartiser et al. (2014) | Statistical software SPSS |
|  | Scott et al. (2017) | Actiware software (version 5.70.1) |
|  | Chen et al. (2015) | Action W2 software and Action 4 software |
|  | Merilahti et al. (2016) | N/A |
|  | Taibi et al. (2013) | Actiware software (version 5.57, Mini-mitter Company, Inc.) and statistical software SPSS |
| **Fourier analysis** | Hauge et al. (2011)  Krane-Gartiser et al. (2014)  Scott et al. (2017) | Online software “Physio Toolkit” (Research Resource for Complex Physiologic Signals) |
| **Sample entropy** | Hauge et al. (2011)  Krane-Gartiser et al. (2014)  Krane-Gartiser et al. (2018)  Scott et al. (2017) | Online software “Physio Toolkit” (Research Resource for Complex Physiologic Signals) |
| **Lempel-Ziv complexity** | Paraschiv-Ionescu et al. (2018) | Additional information presented in paper and in Aboy et al. (1), MATLAB code available in Gao et al. (2) |
|  | Zhang et al. (2018) | Non-commercial activity classification software developed in the FARSEEING EU project and equations presented in paper |
| **Permutation Lempel-Ziv complexity** | Paraschiv-Ionescu et al. (2018) | Additional information presented in paper, MATLAB code available in Bai et al. (3). |
| **Symbolic dynamics** | Krane-Gartiser et al. (2014) | Custom built non available script |

N/A = information not available.

**Supplemental digital content 3.** Correlations and measures of association between the wearable-specific indicators of physical activity behaviour (WIPAB) and intra-individual changes in health-related factors in longitudinal (observational and interventional) studies.

| WIPAB | Reference | Population | Health-related factor | Statistical model | Adjustment of the statistical model | Associations and their direction |
| --- | --- | --- | --- | --- | --- | --- |
| Intensity gradient | Fairclough et al. (2019)  *Intensity gradient at baseline* | Children | BMI z-score  (8-weeks later) | LME (1) | school level clustering, sex, maturation, socio-economic status, baseline health indicator, group allocation, conventional variable (average acceleration) | - |
|  |  |  | Waist-to-height ratio  (8-weeks later) | LME (1) | school level clustering, sex, maturation, socio-economic status, baseline health indicator, group allocation, conventional variable (average acceleration) | ↘ |
|  |  |  | Cardiorespiratory fitness  (8-weeks later) | LME (1) | school level clustering, sex, maturation, socio-economic status, baseline health indicator, group allocation, conventional variable (average acceleration) | - |
|  |  |  | Metabolic syndrome score  (8-weeks later) | LME (1) | school level clustering, sex, maturation, socio-economic status, baseline health indicator, group allocation, conventional variable (average acceleration) | ↘ |
|  |  |  | Health-related quality of life  (8-weeks later) | LME (1) | school level clustering, sex, maturation, socio-economic status, baseline health indicator, group allocation, conventional variable (average acceleration) | - |
| Scaling exponent alpha | Hu et al. (2016)  *Change of activity correlations (alpha) from baseline or the first assessment, alpha at small time scales (< 1.5 hours)* | Elderly with dementia | Change in the Mini-Mental State Examination score (cognition) | LME | - | ↗ |
|  |  |  | Change in the Cornell Scale for Depression in Dementia score (mood) | LME | - | ↘ |
|  |  |  | Increase in the negative affect scale of the Philadelphia Geriatric Centre Affect Rating Scale | LME | - | ↘ |
|  |  |  | Change in the social withdrawal behaviour score of the Multidimensional observation scale for elderly | LME | - | ↘ |
|  | Li et al. (2018)  *Scaling exponent alpha at baseline* | Elderly without dementia at baseline | Decline of global cognitive function | LME (1) | age, sex, education and their interactions with time | ↗ |
|  |  |  |  | LME (2) | 1 + conventional variable (total daily activity level) | ↗ |
|  |  |  | Decline of episodic memory | LME (1) | age, sex, education and their interactions with time | - |
|  |  |  |  | LME (2) | 1 + conventional variable (total daily activity level) | - |
|  |  |  | Decline of working memory | LME (1) | age, sex, education and their interactions with time | - |
|  |  |  |  | LME (2) | 1 + conventional variable (total daily activity level) | - |
|  |  |  | Decline of semantic memory | LME (1) | age, sex, education and their interactions with time | ↗ |
|  |  |  |  | LME (2) | 1 + conventional variable (total daily activity level) | ↗ |
|  |  |  | Decline of perceptual speed | LME (1) | age, sex, education and their interactions with time | ↗ |
|  |  |  |  | LME (2) | 1 + conventional variable (total daily activity level) | ↗ |
|  |  |  | Decline of perceptual orientation | LME (1) | age, sex, education and their interactions with time | ↗ |
|  |  |  |  | LME (2) | 1 + conventional variable (total daily activity level) | ↗ |
|  | Pan et al. (2013)  *Change in local maxima alpha value* | Adults and elderly with Parkinson’s disease | Change in the UPDRS total score | Pearson’s bivariate correlation | - | ↗ |
|  |  |  | Change in the activities of daily living score (UPDRS part II) | Pearson’s bivariate correlation | - | ↗ |
|  |  |  | Change in the motor examination score (UPDRS part III) | Pearson’s bivariate correlation | - | ↗ |

*Note.* The core model consists of an unadjusted model. The covariates, included in the adjusted model, were mostly demographic data (e.g. age and sex). Please refer to the appropriate references for further detail on the included covariates.

LME = linear mixed effects model; cov. = covariates; UPDRS = Unified Parkinson's Disease Rating Scale; (↗) = positive association; (↘) negative association; (-) = no association.

**References**

1. Aboy M, Hornero R, Abásolo D, Álvarez D. Interpretation of the Lempel-Ziv complexity measure in the context of biomedical signal analysis. IEEE Trans Biomed Eng. 2006;53(11):2282-8.

2. Gao J, Cao Y, Tung W-W, Hu J. Multiscale analysis of complex time series: Integration of chaos and random fractal theory, and beyond: John Wiley & Sons; 2007.

3. Bai Y, Liang Z, Li X, Voss LJ, Sleigh JW. Permutation Lempel–Ziv complexity measure of electroencephalogram in GABAergic anaesthetics. Physiol Meas. 2015;36(12):2483.
